# Determinants of unit nonresponse in multi-mode data collection: A multilevel analysis

**DOI:** 10.1371/journal.pone.0215652

**Published:** 2019-04-26

**Authors:** Finaba Berete, Johan Van der Heyden, Stefaan Demarest, Rana Charafeddine, Lydia Gisle, Elise Braekman, Jean Tafforeau, Geert Molenberghs

**Affiliations:** 1 Department of Epidemiology and public health, Sciensano, Brussels, Belgium; 2 Department of Public Health, Epidemiology and Health Economics, University of Liège, Liège, Belgium; 3 Interuniversity Institute for Biostatistics and statistical Bioinformatics, Hasselt University, Diepenbeek, Belgium; 4 Interuniversity Institute for Biostatistics and statistical Bioinformatics, KU Leuven, Leuven, Belgium; Bielefeld University, GERMANY

## Abstract

**Background:**

Multi-mode data collection is widely used in surveys. Since several modes of data collection are successively applied in such design (e.g. self-administered questionnaire after face-to-face interview), partial nonresponse occurs if participants fail to complete all stages of the data collection. Although such nonresponse might seriously impact estimates, it remains currently unexplored. This study investigates the determinants of nonresponse to a self-administered questionnaire after having participated in a face-to-face interview.

**Methods:**

Data from the Belgian Health Interview Survey 2013 were used to identify determinants of nonresponse to self-administered questionnaire (n = 1,464) among those who had completed the face-to-face interview (n = 8,133). The association between partial nonresponse and potential determinants was explored through multilevel logistic regression models, encompassing a random interviewer effect.

**Results:**

Significant interviewer effects were found. Almost half (46.6%) of the variability in nonresponse was attributable to the interviewers, even in the analyses controlling for the area as potential confounder. Partial nonresponse was higher among youngsters, non-Belgian participants, people with a lower educational levels and those belonging to a lower income household, residents of Brussels and Wallonia, and people with poor perceived health. Higher odds of nonresponse were found for interviews done in the last quarters of the survey-year. Regarding interviewer characteristics, only the total number of interviews carried out throughout the survey was significantly associated with nonresponse to the self-administered questionnaire.

**Conclusions:**

The results indicate that interviewers play a crucial role in nonresponse to the self-administered questionnaire. Participant characteristics, interview circumstances and interviewer characteristics only partly explain the interviewer variability. Future research should examine further interviewer characteristics that impact nonresponse. The current study emphasises the importance of training and motivating interviewers to reduce nonresponse in multi-mode data collection.

## Background

Combining various modes of data collection in a single survey has become a common practice in survey research [1, 2]. This approach, referred to as multi-mode data collection (MMDC), can apply to different phases of a survey (pre-contact, main data collection, follow-up) [1, 3–5] and can take different forms. There are four types of MMDC in the main data collection phase [1]:

1. One sample, one time period, one questionnaire
  In this type of MMDC, some respondents of the sample use one mode of data collection while the other respondents use another mode in order to collect the same information. An example of this concurrent mixed-mode design is a paper-and-pencil postal survey offering a web-based option.
2. One sample, one time point, but different modes for different parts of the questionnaire
  This MMDC form refers to the situation when different modes are used for a subset of questions in the questionnaire during a single data collection period. A mix of interview (face-to-face or telephone) and self-administered modes enables to combine the advantages of both methods. For instance, a self-administered questionnaire is used for the more sensitive questions to reduce social desirability and enhance privacy, whereas all other questions are administered by an interviewer.
3. One sample, multiple time points
  The third type of MMDC is used in longitudinal and panel studies where the same respondents are surveyed at multiple time points, using a different mode of data collection from one time point to the other. For instance, the Labour Force Surveys conducted in several countries collect data through face-to-face interviews in the first wave of the survey and through telephone interviews in the second wave, with the same participants.
4. Different samples, different modes
  The last type of MMDC employs different modes of data collection for different populations or subgroups. This type of MMDC is often observed in international or regional comparative studies. Indeed, different countries may have different survey traditions and/or practical constraints, calling for different survey modes for collecting the same information. For instance, in a densely populated country, face-to-face surveys are feasible, but this may not be the case in sparsely populated areas where the data may preferably be collected through distal modes.

The *“One sample*, *one time point*, *but different modes for different parts of the questionnaire”* approach is widely applied in European heath interview and/or health examination surveys, for example in Finland, in the United Kingdom (UK) and in Belgium [6, 7]. In this current study the term MMDC refers to the form “*One sample*, *one time point*, *but different modes for different parts of the questionnaire”*, *an approach inherent to the Belgian Health Survey*.

Although this MMDC design has several advantages (e.g. reduction of measurement error and social desirability), an important and rarely explored limitation is the potential increase in nonresponse due to the time laps that may occur between the administration of the questionnaires [3]. Even if the second questionnaire is administrated immediately after the first, a relatively large number of cases may drop out when switching from one mode of administration to the other. For instance, Sakshaug et al. [8] in their study among alumni from the University of Maryland reported that 26% of those who completed a screening interview never started to complete the subsequent questionnaire. Partial nonresponse in MMDC might also occur because participants consider the second part of the questionnaire as non-relevant, non-essential or non-compulsory to the first, or even as an independent survey.

Nonresponse is a major concern in population health surveys, since it is a threat to the validity of the results [9, 10]. In MMDC, when a participant fails to complete all the stages of data collection, for example by not completing the self-administered questionnaire (SAQ) after having participated in a face-to-face interview, this nonresponse leads to missing data for all the items included in the SAQ. The main implications of this partial nonresponse are a decrease in statistical power, a larger standard error and a nonresponse bias [11].

The selection mechanism of partial nonresponse in MMDC shows some similarities with what can be observed in second stage nonresponse. In a second stage recruitment, participants of one survey are asked to participate in yet another survey [12]. In both situations, data collection takes place in a population that has already been willing to participate in the survey.

To date, numerous studies have explored determinants of nonresponse in a Health Interview Survey (HIS). A relationship has been shown with societal factors and characteristics of the survey design [13], characteristics of the sampled persons [11, 13–16], characteristics of the interviewer [13, 17–20], area characteristics [14, 20, 21] and participant-interviewer interaction [13]. However, most of these studies have explored initial nonresponse. For instance, findings regarding nonresponse in the Belgian HIS (BHIS) concern initial nonresponse, are restricted to the household level [22, 23] and only one of them assessed interviewer effects [23].

Much fewer studies have investigated the selection mechanism in second-stage nonresponse in health surveys. Among these, second stage nonresponse was shown to be related to the characteristics of the sampled persons (e.g. socioeconomic status and nationality) [11, 12]. No study has yet assessed the determinants of partial nonresponse in MMDC population health surveys.

This study aims to investigate nonresponse in MMDC using the 2013 Belgian Health Interview Survey (BHIS 2013). The BHIS questions are administered through a face-to-face interview followed by a SAQ. This is of particular interest due to the increasing nonresponse to the SAQ in the BHIS over the years, especially in 2013. Knowing the determinants of SAQ nonresponse is useful for implementing measures to reduce this nonresponse in future surveys.

The purpose of this study is therefore to examine whether there are systematic differences between interviews regarding nonresponse to the SAQ in the BHIS, and whether these differences can be explained by participant characteristics, interview circumstances and interviewer characteristics.

## Methods

### Ethics statement

The BHIS 2013 was carried out in line with the Belgian privacy legislation. Ethical approval for the data collection was given by the Ethical Committee of Ghent University Hospital on October 1, 2012. There was no formal written and signed consent foreseen as participation was considered as consent. The selected households where notified about the survey, its practical organization, the institution in charge, the commissioners of the survey and its content via a letter and an information leaflet personally addressed to them. It was also clearly stipulated in the letter and the leaflet that participation is voluntary. All data were fully anonymized before their access. The BHIS data are personal data that contain sensitive information about the respondents. They are considered as coded data where the law of public statistics applies. In order to comply with the current regulations in this domain, access to these data is only possible through a request to the Health Committee of the Data Protection Authority. Further information regarding the survey and the data access procedure can be found here: http://www.healthsurvey.be.

### Study population and data

The BHIS is a cross-sectional nationwide household survey on health status, medical consumption and lifestyle habits of the Belgian population. It has been organised every 4 to 5 years since 1997. The participants are selected from the national population register through a multistage stratified sample of the population. For this study we used data from the BHIS 2013. In 2013, 9,561 households were invited to participate in the survey. Among them, 5,049 participated, 3,801 refused to participate, 497 were not contactable and 304 were not eligible. So, the response rate was 54% (i.e., 5,049/(3,801+5,049+497)) and the participation rate was 57% (i.e., 5,049/(3,801+5,049)), at the household level. The detailed methodology of the BHIS is described elsewhere [24]. Data collection is done by means of computer-aided personal interviews (CAPI) and a SAQ at the end of the CAPI interviews. Proxy interviews were conducted for participants younger than 15 years, for those not capable of responding personally, for those who refused to respond personally or for those who were not contactable for at least 3 months. Proxy participants were not eligible to complete the SAQ.

**Fig 1:**
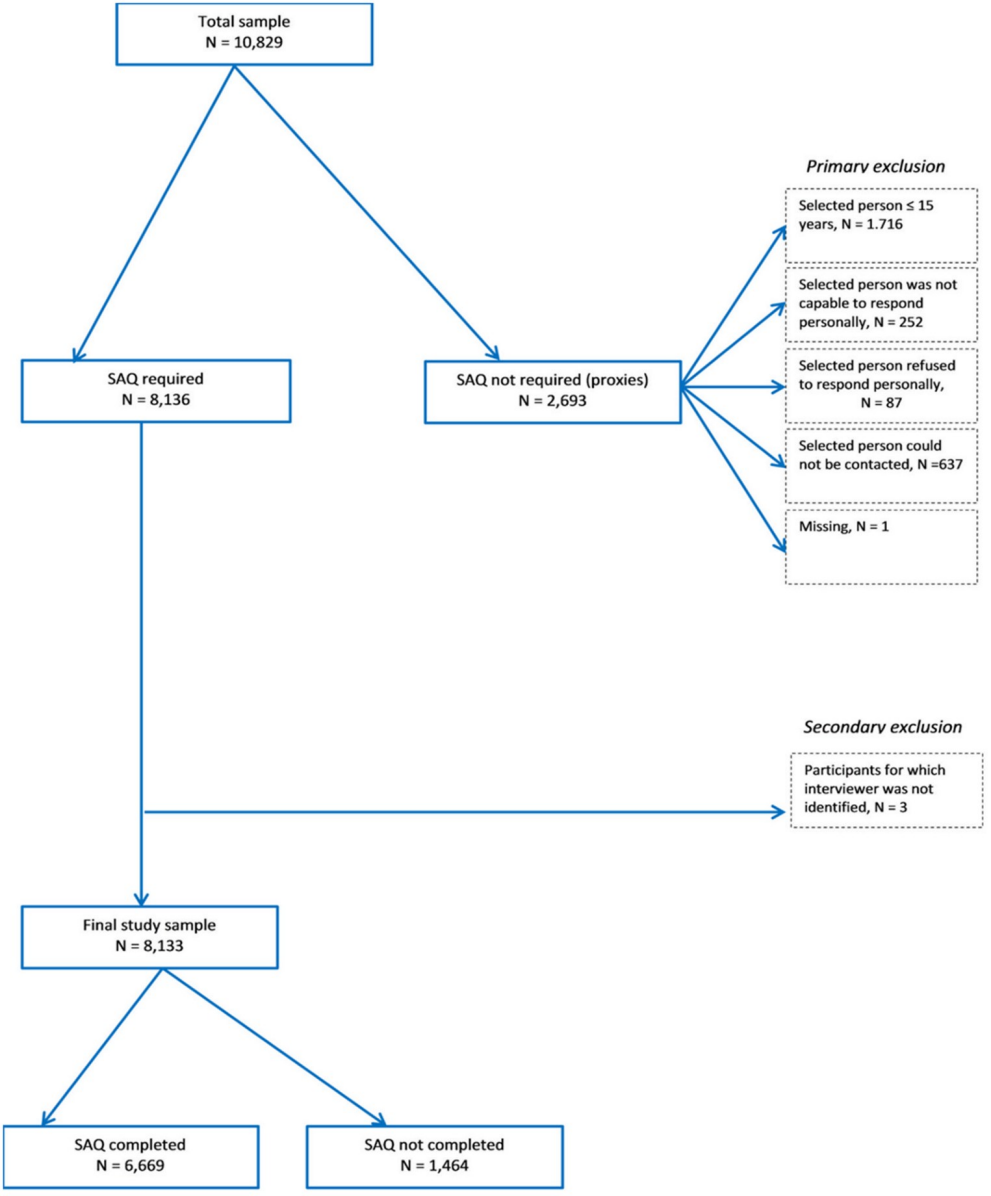
Flow diagram of the number of participants included in the study.

The total sample of the BHIS 2013 was 10,829 individuals interviewed by 183 trained interviewers. As in most face-to-face interview methodology, for logistical reasons, interviewers were not randomly allocated to municipalities (primary sampling unit). In the BHIS fieldwork, one interviewer may work in more than one municipality and sampled cases in one municipality may be assigned to more than one interviewer as municipalities are divided into groups of 50 interviews. However, such situations were rather rare as 86% of the interviewers worked in only one single municipality and as 71% of the municipalities had only one operating interviewer. Completing the SAQ was required for 8,136 individuals and has been completed by 6,669 respondents (82%). After excluding individuals for whom complete information on the interviewer was not available, the final sample size for this study is 8,133 individuals, Fig 1.

### Potential determinants of nonresponse

Determinants were chosen based on their predictive capacity for nonresponse or because they were related to nonresponse in other studies [10, 11, 25]. We considered determinants at three levels: the participant, the interview and the interviewer.

Variables selected at the participant level were gender (male/female), age group (15–34, 35–54 and ≥ 55 years), nationality (Belgian/European/Non-European), educational level (primary or no degree/secondary inferior/secondary superior/superior), household size (1 to 4+ members), household income level (quintiles), region of residence (Flanders/Brussels/Wallonia) and having a chronic health condition (yes/no). The presence of chronic conditions (suffering from one or more long-standing illnesses, chronic conditions or handicaps) was used as proxy for health status.

Information on the interview circumstances were the duration of the interview (short/average/long) and the quarter of the year in which the interview took place. The duration of the interview is assumed to negatively influence the probability of completing the SAQ. The longer the duration, the higher the probability of not completing the SAQ at the end of the CAPI interview. Also the quarter of the year in which the interview took place may influence nonresponse to the SAQ.

The included interviewer-related variables were gender (male/female), age group (20-44/45-64/>64 years), educational level (primary/secondary inferior/ secondary superior/superior/unknown), professional status (active/unemployed or retired/others or unknown), prior experience with interview surveys (yes/no), number of interviews achieved per interviewer (low/average/high /very high). The number of interviews achieved per interviewer is based on the total interviews each interviewer completed during the HIS project. It was included in order to evaluate the impact of the interviewer’s workload on nonresponse rate.

An additional and important factor which can also influence the response rate to SAQ is the way the interviewer presented the SAQ to respondents, either as a required questionnaire to be completed or as a supplementary (‘extra’) questionnaire which respondents could, or could not complete. While we do not have the information to verify this hypothesis, it remains an important aspect relating to nonresponse in MMDC.

### Statistical analyses

Weighted proportions and means were calculated to present the characteristics of the population.

A multilevel logistic regression model was used to assess the determinants associated with nonresponse. This approach is advocated and implemented in various cross-sectional studies to disentangle effects associated with each level [25–27]. The survey design and the related within-cluster dependence make ordinary regression modelling inappropriate, so the use of multilevel models is recommended to accommodate such dependence [28]. The interviewer level is therefore included as a random effect to account for possible correlation within clusters [29]. The reasoning behind adding the characteristics of the interviewer as fixed effects is to understand to which extent they explain interviewer variability in nonresponse rate.

To obtain the best fitting and most parsimonious model for the data set and research questions, the 2-levels Generalised Linear Mixed Models with Random intercepts only is used. Individuals (level 1 units) are modelled as nested within interviewers (level 2 units). In addition to the main effects, some interaction effects (between participant and interviewer age group; participant and interviewer gender) were also studied. The model building process was done in 4 steps, as follows:

Step 1: Model 0 (empty model)
  In the first step, the model was fitted without covariates at any level (i.e. only random effects for the intercept) to assess whether there was a significant variation at the interviewer level [26, 27].
Step 2: Model 1
  Participant characteristics were included as fixed effects. The results of model 1 indicate the relationship between the level 1 predictors and the nonresponse to the SAQ.
Step 3: Model 2
  The interview circumstances were added as fixed effects in order to assess to what extent their addition altered the relationships observed in the previous model and to identify the relationship between these predictors and the outcome.
Step 4: Model 3
  The interviewer characteristics were added to model 2. The result of this final model allows to identify the relationship between the level 2 variables and the outcome.

No survey settings were used in the multilevel analyses. However, all variables used in the calculation of the survey weights were included as covariates in the final model.

Across these models, we then compared the estimated interviewer variance components. To allow for this comparison, a rescaling procedure was performed to take into account the implicit scale changes in logit model [30]. We estimate the multilevel logit model with the Stata command xtmelogit and rescale parameters by means of the command meresc developed by Enzmann and Kohler [31].

Variance components were tested against zero using the likelihood ratio test, asymptotically distributed as a mixture of a Chi-Squared with zero and a Chi-Squared with one degree of freedom [32]. The intraclass correlation coefficient (ICC) was used to measure how much of the total variation in the probability of being a nonrespondent is accounted for by the interviewers [26]. The proportional change in variance (PCV) between the initial model and the model with more terms was also calculated [33, 34]. Associations were expressed as odds ratios (OR) together with their 95% confidence interval (CI). Likelihood ratio (deviance) tests were conducted to compare the relative fit of the different models [26]. The difference in deviance of two nested models follows a chi-square distribution with degrees-of-freedom equal to the number of additional parameters in the larger model [35]. All statistical analyses were carried out using SAS v.9.3 and Stata 14.

## Results

### Background characteristics of BHIS participants and interviewers

The eligible sample consisted of 8,133 participants aged 15 years and older, of which 1,464 (18%) did not complete the SAQ.

Table 1 presents the characteristics of participants and interviewers. BHIS participants were more likely female, highly educated and without chronic conditions. Additional participant characteristics can be found in Table 1.

**Table 1:** Characteristics of the study population, interview circumstances and interviewers’ characteristics, Belgian Health Interview Survey, 2013 (weighted percentages).

	%	N
**Participant characteristics (N = 8,133)**		
***Gender***		
Male	48.1	3867
Female	51.9	4266
***Age group (years)***		
15–34	27.1	2106
35–54	35.7	2810
≥ 55	37.2	3217
***Nationality***		
Belgian	90.3	7042
European	5.8	690
Non-European	3.8	396
Missing	0.04	5
***Education level***		
Primary/No degree	10.0	860
Secondary inferior	12.9	1151
Secondary superior	34.0	2602
Superior education	42.2	3406
Missing	0.9	114
***Chronic conditions***		
Yes	29.0	2562
No	70.9	5570
Missing	0.00	1
***Household size***		
1	19.1	1701
2	33.6	2751
3	17.7	1398
4+	29.6	2283
***Household income***		
Quintile 1	18.3	1685
Quintile 2	15.9	1243
Quintile 3	17.9	1381
Quintile 4	19.6	1422
Quintile 5	19.2	1455
Missing	9.1	947
***Region***		
Flanders	58.4	2831
Brussels	9.7	2062
Wallonia	31.9	3240
**Interview circumstances**		
***Length of interview***		
Short *(facetime ≤ 13’)*	25.1	1829
Average *(13’ < facetime ≤ 37’)*	49.5	4091
Long *(facetime > 37’)*	25.4	2213
***Quarter of interview***		
Quarter 1	24.0	1367
Quarter 2	24.6	1591
Quarter 3	25.5	2123
Quarter 4	25.8	3052
**Interviewer characteristics (N = 183)**		
***Gender***		
Male	62.1	110
Female	37.9	73
***Age group (years)***		
20–44	19.9	45
45–64	53.9	96
> 64	26.2	42
***Education level***		
Secondary inferior/Secondary superior	34.7	65
Superior education	54.7	95
Unknown	10.6	23
***Professional status***		
Active	54.3	90
Unemployed/Retired	30.9	60
Other/Unknown	14.7	33
***Prior experience with the HIS***		
Yes	87.9	141
No	12.1	42
***Number of interviews performed by interviewer***		
Low *(count < 47)*	46.8	88
Average *(47* ≤ *count < 60)*	24.5	41
High (60 ≤ *count < 98)*	23.2	36
Very high *(≥ 98)*	5.5	18

About half of the interviews were conducted in a time period running between 13 and 37 minutes. The average duration of an interview was 56 minutes (SD approx. 2.5 minutes). The number of interviews carried out was slightly higher in the last two quarters of the year. One hundred and eighty-three interviewers were involved in this study. Interviewers were more often male (62.1%), aged between 45–64 years (53.9%) and highly educated (54.7%). More than 50% of the interviewers held another job and 87.9% of them were experienced interviewers. The average number of interviews carried out per interviewer (in a 12-month period) was 94.5 (SD ± 1.1), the minimum was 1 and the maximum was 565 interviews. More than half of the interviewers performed at least 47 interviews (Table 1).

### Determinants of nonresponse to SAQ

Table 2 reports the estimated random effect parameters for the different model specifications, as well as model fit statistics. Model 0 (empty model) captures how much of the total variation in the probability of not completing the SAQ is attributable to interviewers. According to the ICC, interviewer effects were very high. Up to 47% of the variability in the nonresponse was accounted for by the interviewers, leaving 53% of the variability to be accounted for by participant and others unexplored factors. Based on the likelihood ratio test (against the logistic model), one concludes that the interviewer level is required and that there was a significant variability in nonresponse to SAQ between interviewers.

**Table 2:** Estimated variance components, intraclass correlations and model fit statistics for different specifications of the multilevel models for nonresponse to self-administered questionnaire, Belgian Health Interview Survey, 2013.

Model	0	1	2	3
	Intercept only	With respondent characteristics	With interview circumstance	With interviewer characteristics
**Variance components**				
*Not scaled*:				
$\sigma_{e}^{2}(individual\;level)$	3.290			
$\sigma_{u}^{2}\;(interviewer\;level)$	2.869	2.663	2.879	2.593
*Scaled*				
$\sigma_{e}^{2}(individual\;level)$		3.072	3.025	2.898
$\sigma_{u}^{2}\;(interviewer\;level)$		2.486	2.654	2.284
**ICC**_interviewers_	0.466	0.447	0.467	0.441
**PCV**		13.3%	7.5%	20.4%
**Model fit**				
Log likelihood	-3099.214	-2442.017	-2416.269	-2410.129
LR-Test *vs*. logistic model (p-value of LR test)	1469.44 (0.000)	802.09 (0.000)	807.92 (0.000)	760.09 (0.000)
LR-Test against previous model (df; p-value of LR test)		1314.394 (7; 0.000)	51.496 (2; 0.000)	12.280 (1; <0.005)

As shown in Table 2, adding participant characteristics to the model (model 1) decreased the interviewer variance and explained 13.3% of this variance in the empty model. The likelihood ratio test (against previous model) reveals that adding participant characteristics has significantly improved the model (p<0.0001).

The additional inclusion of interview circumstances variables increased the interviewer variance (model 2) even after rescaling. Together with participant characteristics, interview circumstances and interviewer characteristics explained 20% of the interviewer variance (model 3). Even after controlling for interviewer characteristics, the interviewer effects remained significant.

Based on the likelihood ratio tests, model 3 was the best fitting model and therefore parameter estimates from this model were used to explore the determinants of SAQ nonresponse.

Table 3 presents the rescaled estimated odds ratios and their 95% CI for the model specifications. As stated above, we comment only on the final model.

**Table 3:** Odds ratios (and 95%CI) of SAQ nonresponse based on the multilevel analysis model, Belgian Health Interview Survey, 2013 (rescaled estimates).

*	Model 0 (Empty model)	Model 1	Model 2	Model 3^a^
		OR (95% IC)	OR (95% IC)	OR (95% IC)
**Fixed effects at individual-level**				
***Gender***				
Male		1.11 (0.96–1.28)	1.11 (0.96–1.28)	1.10 (0.96–1.27)
Female		1.00	1.00	1.00
***Age group (years)***				
15–34		1.08 (0.90–1.30)	1.06 (0.88–1.28)	1.06(0.88–1.28)
35–54		1.00	1.00	1.00
≥ 55		0.77 (0.65–0.93)*	0.77 (0.64–0.92)*	0.78 (0.65–0.93)*
***Nationality***				
Belgian		1.00	1.00	1.00
European		1.39 (1.10–1.77)*	1.40 (1.11–1.78)*	1.40 (1.11–1.77)*
Non-European		1.58 (1.18–2.13)*	1.60 (1.19–2.15)*	1.59 (1.19–2.12)*
***Education level***				
Primary/No degree		1.95 (1.50–2.54)*	1.91 (1.47–2.48)*	1.89 (1.46–2.44)*
Secondary inferior		1.10 (0.86–1.40)	1.06 (0.83–1.35)	1.05 (0.83–1.34)
Secondary superior		1.17 (0.97–1.41)	1.15 (0.95–1.37)	1.14 (0.95–1.37)
Superior education		1.00	1.00	1.00
***Region***				
Flanders		1.00	1.00	1.00
Brussels		2.38 (1.45–3.93)*	2.27 (1.37–3.78)*	2.10 (1.29–3.42)*
Wallonia		1.75 (1.05–2.91)*	1.72 (1.02–2.89)*	1.73 (1.06–2.83)*
***Chronic conditions***				
Yes		1.17 (0.99–1.38)	1.20 (1.02–1.42)*	1.20 (1.02–1.41)*
No		1.00	1.00	1.00
***Household income***				
Quintile 1		2.06 (1.61–2.64)*	2.05 (1.60–2.63)*	2.02 (1.59–2.58)*
Quintile 2		1.76 (1.36–2.27)*	1.78 (1.38–2.30)*	1.76 (1.36–2.26)*
Quintile 3		1.09 (0.84–1.41)	1.10 (0.85–1.43)	1.10 (0.85–1.42)
Quintile 4		1.00	1.00	1.00
Quintile 5		0.92 (0.70–1.21)	0.91 (0.69–1.19)	0.91 (0.70–1.19)
**Fixed effects at interview-level**				
***Quarter of interview***				
Quarter 1			1.00	1.00
Quarter 2			1.66 (1.26–2.20)*	1.65 (1.26–2.17)*
Quarter 3			2.06 (1.56–2.71)*	2.06 (1.55–2.66)*
Quarter 4			2.43 (1.87–3.15)*	2.39 (1.85–3.08)*
***Length of face-to-face interview***				
Short *(facetime ≤ 13’)*			1.19 (0.97–1.46)	1.19 (0.97–1.45)
Average *(13’ < facetime ≤ 37’)*			1.00	1.00
Long *(facetime > 37’)*			1.00 (0.83–1.20)	1.00 (0.83–1.19)
**Fixed effects at interviewer level** ^b^				
***Number of interviews performed by interviewer***				
Low *(count < 47)*				3.30 (1.60–6.79)*
Average *(47* ≤ *count < 60)*				1.00
High (60 ≤ *count < 98)*				3.08 (1.37–6.92)*
Very high *(≥ 98)*				(1.43–10.22)*

Observations: 7,089 respondents, 183 interviewers, ICC = intraclass coefficient of correlation; PCV = proportional change in variance. The PCV expresses the change in the interviewer’s level variance between the initial model and the model with more terms.

^a^ Best fitting model;

^b^ Only variables that were significantly related to nonresponse after adjusting for all other variables.

*p value <0.05

After adjusting for all other variables, participant characteristics were found to be important determinants of nonresponse. Participants aged 35 to 54 years were more likely not to complete the SAQ compared to those aged 55 years and older. Furthermore, compared to their counterparts, non-Belgian participants, who are lower educated, who suffer from chronic conditions and who belong to a lower income household had higher odds of not responding to the SAQ. As well, significant differences in nonresponse were observed between participants living in Brussels (OR = 2.10, 95% CI 1.29–3.42) and in Wallonia (OR = 1.73, 95% CI 1.06–2.83) compared to those living in Flanders.

Concerning the circumstances of the interview interviews performed during the second, third and fourth quarter of the year were significantly associated with higher odds of nonresponse, with respectively (OR = 1.65, 95% CI 1.26–2.17); (OR = 2.06, 95% CI 1.55–2.66); (OR = 2.39, 95% CI 1.85–3.08) compared to those performed in the first quarter of the year. Furthermore, participants whose interviews took a short time (maximum 13 minutes) had significantly higher odds of being nonrespondent for the SAQ than those with an interview length of average duration (i.e. between 13 and 37 minutes). However, this difference is not statistically significant, but is close to reaching the level of significance.

In the full model, interviewer characteristics were no longer significantly related to nonresponse. Only the number of interviews performed per interviewer remained significant. Compared to those who carried out an average number of interviews, interviewers who performed a low, a high and a very high number of interviewers reported higher nonresponse rates to SAQ.

## Discussion

This study investigated whether there were systematic differences between interviewers regarding nonresponse to the SAQ in the BHIS, and whether these differences could be explained by participant characteristics, interview circumstances and interviewer characteristics.

### Variability between interviewers

Substantial variability between interviewers was found. Almost half of the total variance in nonresponse was found at the interviewer level, which confirms that there are systematic differences between interviewers for participants’ nonresponse to the SAQ. However, in most interview surveys, interviewers work in a limited geographical area and, as people from certain areas may be more or less likely to cooperate, a significant interviewer effect may simply indicate an existing neighbourhood effect [16, 36]. To test whether this variability did not mainly reflect area variability, municipalities were included as random factors in the empty model. These municipalities differed significantly in nonresponse rate. However, the three-level model with municipalities as level 3 units showed a non-significant area effect, implying that, for this study, a simpler two-level model was indeed sufficient, even after the inclusion of explanatory variables. These results are consistent with previous studies [16, 25].

It is striking that the interviewer effect in this study is much more pronounced compared to other studies that investigated interviewer effects for nonresponse or non-contact in surveys [16, 25, 36–38]. This difference might simply indicate that the interviewer role in MMDC is more relevant than it would be in initial nonresponse. Indeed, in MMDC the interviewer has to reinforce the participant’s motivation to continue with the SAQ. Maintaining participant motivation depends on the way in which the interviewer presents the whole survey and emphasises that both questionnaires, the CAPI and the SAQ, are necessary parts of the same survey. Furthermore, the willingness to continue with the SAQ may also rest on the interaction with the interviewer during the face-to-face interview. Finally, the interviewer’s lack of rigour in the follow-up of the SAQ can influence its missingness. Therefore, further efforts to reduce nonresponse to SAQ should first focus on reducing interviewer variability.

### Influence of participant characteristics

Several characteristics of the participants were identified as independently linked to nonresponse to SAQ, suggesting that nonresponse to SAQ was not random.

A higher nonresponse was found among participants younger than 55 years old. This group may have less time to take part in the face-to-face interview and to complete the SAQ the same day due to work obligations and family commitments. This result is in agreement with previous studies [12, 39]. Furthermore, in accordance with other studies on initial nonresponse [9, 15, 40] or second stage nonresponse [11, 12, 39, 41], nonrespondents were more likely to be less educated, non-Belgian and belonging to a lower income household. Higher nonresponse among non-Belgians participants, especially among non-Europeans might be related to a lower socio-economic status. The SAQ is a self-administered written questionnaire in French, Dutch, German or English, which probably favours non-Belgians who are well-integrated or well educated. Earlier studies on initial nonresponse in BHIS at the household level reported similar results [22, 42]. Unfortunately, a rigorous comparison with these studies is not feasible due to methodological differences. Nevertheless, it is possible that the mechanisms of initial nonresponse in BHIS were not very different from those found in this study with regard to the background characteristics of the participants, since the characteristics of the reference person are closely related to those of other household members.

Participants living in Brussels were more likely not to respond to the SAQ compared to those living in Flanders. This might be due to the fact that Brussels has a higher proportion of people with lower socio economic status. This population has more difficulties with the language/comprehension and is less interested in participating in SAQs where they have to read the questions themselves. This finding was in line with another study [40]. Furthermore, Brussels is predominantly an urban area and the finding of a higher nonresponse in Brussels is consistent with other studies that reported lower survey response rates in urbanized settings, relative to rural and less urbanized areas [43].

The results support the hypothesis that the mechanisms of nonresponse to SAQ are not very different from those found in initial nonresponse, as confirmed by earlier studies [11, 12]. Further efforts to reduce nonresponse to the SAQ in BHIS should take into account participants who were more likely to be nonrespondents, i.e. those of working age, those with lower socio-economic status and those living in Brussels.

### Influence of interview circumstances

Interview circumstances were found to be important determinants of nonresponse. However, it must be recognised that there were some outliers in the face-to-face interview duration. The minimum length was 0 minutes and the maximum was 24 hours, which are unrealistic values. Obviously, this might be due to technical issues in the CAPI application. Sensitivity analyses were conducted with and without outliers, but the difference was negligible. Therefore, these outliers were kept in the analyses.

Participants interviewed during the last quarters of the year have higher odds of being nonrespondents to the SAQ than those interviewed during the first quarter of the year. This might be a result of the BHIS fieldwork. Indeed, to compensate for the BHIS non-participating rate, the number of interviews to be carried out by the interviewer is increased from one quarter to the next, which leads to an increase in interviewer workload for the last quarters. The aim of the working method is to ensure reaching the number of planned interviews by the end of the 12-month fieldwork period. Japec (2008, cited by [44]) mentioned that interviewer workload is generally seen as a negative influence on their performance. Other authors argue that increased workload give interviewers less time to attempt contact during the most productive times [45]. Similarly, it is possible that in the BHIS, interviewers with heavier workload have less time to wait for the interviewees to complete the SAQ and are therefore tempted to leave the questionnaire to the participant and do not return to get it.

It is striking that a short interview duration yields higher odds of being a SAQ nonrespondent, which was the opposite of what was expected. An explanation of this result could be that short duration interviews reflect the poor motivation of respondents and their lack of interest to continue with a SAQ.

### Influence of interviewer characteristics

We observed significant and important interviewer effects. After adjusting for participant characteristics and interview circumstances, none of the interviewers’ sex, age, education, professional status and experience explained this variation. These characteristics are thus not a major source of interviewer effects. Only the number of interviews performed per interviewer was significantly related to SAQ nonresponse. Compared to those who carried out an average number of interviews, interviewers who performed a high or very high number of interviews recorded a high proportion of nonresponse for the SAQ. Another surprising result was that interviewers who carried out a few interviews also have higher odds of nonresponse compared to those who carried out an average number of interviews. This might partially be attributed to their characteristics. In fact, although they are all experienced interviewers, almost two thirds of the interviewers who performed a low number of interviews held another job compared to barely a third of those who carried out an average number of interviews. Combining their regular employment with the job of interviewer leads to tighter time constraints and organisation.

These findings are in line with previous studies that found that interviewer background characteristics (sex, age, education, socioeconomic status, work experience) were not good predictors of interviewer level variance [10, 16, 35], whilst respondent-interviewer interaction has found to be a good predictor. For instance, Pickery and colleagues [16], emphasised the importance of the first positive interview experience for the response in the subsequent waves of a panel survey. Another author showed that a positive survey experience enhances the chance that people will participate in subsequent surveys, whereas those without such an experience are less likely to participate [46]. Although this study is not a panel survey, one can hypothesise that the experience of participants during the face-to-face interview and their interaction with the interviewer may affect their propensity to complete the subsequent SAQ. Moreover, another study [10] reported that interviewer level response rate can be predicted by interviewer attitude towards the interviewer’s role. Interviewers who were more inclined to favour persuading the participants had a higher response rate, while those who were more inclined to favour acceptance of refusals and not persuading the participants had a lower response rate. Furthermore, other explanations of the higher nonresponse for the SAQ could be of course fatigue or time constraints for respondents and interviewers. However, we suspect that interviewers presented the SAQ to participants as a “second stage recruitment”. Although not scheduled as such, it looks that a substantial portion of interviewers thought and introduced the SAQ as a supplementary (‘extra’) questionnaire which respondents could or not complete. So, after completing the CAPI, respondents “were asked” if they were willing to complete the SAQ. This was of course not the idea, nor the correct procedure. Interviewers should have presented the survey as consisting of two necessary parts and not as a main core interview (CAPI) with complementary questions (SAQ).

Even after taking into account interviewer characteristics, a significant part of interviewer variability remained unexplained. This might be because the relevant interviewer characteristics were not available in our data. Future investigation should include, for example, interviewer attitude and motivation [19, 47], participant-interviewer interaction and interviewer expectation [13], interviewer attitude regarding the persuasion of reluctant respondents [10, 44], and interviewer own behaviour regarding data collection requests [18].

### Strengths and limitations

The major strengths of this study include its relatively large sample of participants and interviewers and the use of multilevel analysis. The large sample size for both participants and interviewers increased the ability to detect specific interviewer effects. A multilevel analysis allowed to separate the potential sources of variability and to explore clustering effects.

Conveniently, an interpenetrated design is used when interviewers are allocated at random to participants [48, 49]. However, in practice, randomly allocating interviewers to participants is expensive and difficult to organise [44, 50]. Consequently, much of the literature on interviewer effects consists of either telephone surveys with small numbers of interviewers or face-to-face surveys without interpenetrated designs [51]. In this study, for practical reasons, interviewers are not randomly assigned to participants. Without these interpenetrating designs, the interviewer and participants explanatory variables are correlates and interviewer intraclass correlation no longer estimates interviewer effects only [50]. This is a limitation of the current study. However, this difficulty has been overcome statistically by adding participant characteristics and interviewer characteristics in two separate steps as recommended by several authors [44, 50]. This therefore allowed distinguishing participants and interviewer effects. Interviewer effects remained significant even after controlling for participant characteristics.

A number of additional study limitations need to be acknowledged. Firstly, the clustering effect at the household level was not explored. However, earlier studies have shown that the impact of intra-household effect was negligible [22, 42]. Secondly, even if the findings are relevant for Belgium, they are restricted to specific situations considered in this study. Therefore, the results cannot automatically be extrapolated to different settings or other surveys. Further research investigating different situations and data structures should be carried out to confirm our results.

The impact of SAQ nonresponse on BHIS results might potentially be important as it took place among participants who might already be a selective sample from the general population. While the analysis of this impact is beyond the present study, an interesting idea for future research would be to assess the consequences of this nonresponse on the estimates.

## Conclusions

To the best of our knowledge, this is the first study investigating partial nonresponse in MMDC. The results contribute to a better understanding of the nonresponse in such a design. The findings highlight that interviewers do play a crucial role in nonresponse to the SAQ in the context of MMDC. Unfortunately, the interviewers’ characteristics included in our analyses do not contribute to explain this variability.

The inter-interviewer variability is partially explained by the participant characteristics, interview circumstances and interviewer characteristics involved in the analyses. However, further interviewer characteristics and interviewer-participant interaction should be considered for future research. Nevertheless, this study has a major implication for survey researchers since it provides some support to reduce SAQ nonresponse. Although some of the determinants of nonresponse are beyond the control of the researchers (e.g. participant characteristics), they might be highly useful regarding survey implementation in order to reduce nonresponse.
